# A Combination of Compositional Index and Genetic Algorithm for Predicting Transmembrane Helical Segments

**DOI:** 10.1371/journal.pone.0021821

**Published:** 2011-07-26

**Authors:** Nazar Zaki, Salah Bouktif, Sanja Lazarova-Molnar

**Affiliations:** 1 Intelligent Systems, Faculty of Information Technology, United Arab Emirates (UAE) University, Al-Ain, United Arab Emirates; 2 Software Development, Faculty of Information Technology, United Arab Emirates (UAE) University, Al-Ain, United Arab Emirates; Università di Napoli Federico II, Italy

## Abstract

Transmembrane helix (TMH) topology prediction is becoming a focal problem in bioinformatics because the structure of TM proteins is difficult to determine using experimental methods. Therefore, methods that can computationally predict the topology of helical membrane proteins are highly desirable. In this paper we introduce TMHindex, a method for detecting TMH segments using only the amino acid sequence information. Each amino acid in a protein sequence is represented by a Compositional Index, which is deduced from a combination of the difference in amino acid occurrences in TMH and non-TMH segments in training protein sequences and the amino acid composition information. Furthermore, a genetic algorithm was employed to find the optimal threshold value for the separation of TMH segments from non-TMH segments. The method successfully predicted 376 out of the 378 TMH segments in a dataset consisting of 70 test protein sequences. The sensitivity and specificity for classifying each amino acid in every protein sequence in the dataset was 0.901 and 0.865, respectively. To assess the generality of TMHindex, we also tested the approach on another standard 73-protein 3D helix dataset. TMHindex correctly predicted 91.8% of proteins based on TM segments. The level of the accuracy achieved using TMHindex in comparison to other recent approaches for predicting the topology of TM proteins is a strong argument in favor of our proposed method. **Availability:** The datasets, software together with supplementary materials are available at: http://faculty.uaeu.ac.ae/nzaki/TMHindex.htm.

## Introduction

A biological membrane or biomembrane is an enclosing or separating membrane that acts as selective barricade within or around a cell in which cells may maintain specific chemical or biochemical environments. Membrane proteins play key roles in biological systems as pores, ion channels and receptors. Being important in intracellular communication and coordination, membrane proteins may serve as good drug targets. A biological membrane is usually spanned by a TM protein which makes it an important target of both basic science and pharmaceutical research [1]. The major category of TM proteins is the 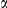-helical proteins. This protein category constitutes roughly 30% of a typical genome and is usually present in the inner membranes of bacterial cells, the plasma membrane of eukaryotes, the outer membrane of Gram negative bacteria or mitochondrial membranes. 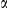-helical transmembrane proteins are involved in a wide range of important biological processes such as cell signaling, transport of membrane-impermeable molecules, cell-cell communication, cell recognition and adhesion. Since many TMHs are also prime drug targets, it has been estimated that more than half of currently commercialized drugs target membrane proteins [2]. Therefore, the prediction of TMHs could play an important role in the study of membrane proteins. The importance of this role is emphasized by the lack of high-resolution structures for such proteins. Thus, the total number of transmembrane proteins in the Protein Data Bank (PDB) [3] is limited, comprising 1% of available structures [4], [5]. Knowledge of the TMH topology can help in identifying binding sites and infer functions for membrane proteins. However, because membrane proteins are hard to solubilize and purify, only a very small amount of membrane proteins have experimentally determined structure and topology. This has motivated various computational methods for predicting the topology of membrane proteins [6]. These methods are important applications in genome analysis, and can be used to understand the global trend in membrane protein evolution.

A computational method is usually considered successful if it does not only predict individual TMHs, but rather attempt to predict the full topology of the protein [7]. To this end, in the last two decades, researchers have developed a battery of successively more powerful methods for predicting TMH. This development can be broken into three main categories. In the first category, early TMH prediction methods were based on experimentally determined hydropathy indices of hydrophobic properties for each residue in the protein sequence. Examples of this category include TOP-Pred [8], DAS-TMfilter [1] and SOSUI [9] which are among the most reliable methods in providing descriptive information about TMHs. These methods use hydrophobicity analysis alone and therefore, they can not predict TMHs with length greater than 25 residues [10]. The recent high-resolution structures production of helical membrane proteins revealed that TMH could have a wide length distribution of more than 25 residues.

In the second category, further accuracy was achieved by employing probabilistic approaches such as Hidden Markov Models (HMMs). In this case the actual biological structural knowledge was incorporated into the model's architecture in order to increase its prediction power. Methods such as HMM-TOP [11], TMHMM [12], THUMBU [13] and Phobius [14], allowed researchers to predict reliable integral membrane proteins in a large collection of genome. However, HMM based methods are considered computationally expensive since they involve multiple sequences alignments, calculation of the profile HMM topology and parameterization, and training via expectation maximization. Moreover, the HMM based methods are unable to correctly predict TMHs shorter than 16 residues or longer than 35 residues [10]. As for distantly related protein sequences, a profile alignment may not be possible if, for example, the sequences contain shuffled domains.

In the third category, additional accuracy was gleaned by leveraging machine learning techniques such as neural networks, support vector machines and k-nearest neighbor. Examples of this category include PHD [15], MemBrain [10] and MEMSAT-SVM [2]. Despite their success, the feature extraction step in the machine learning based techniques is often computationally expensive since they also involve multiple sequences alignments. Therefore, a simple and general feature extraction algorithm that do not require sequence alignments is desirable.

Numerous methods have also been developed to study secondary structure assignment [16]–[18]. Pylouster et al. [19] have recently studied the influence of the assignment on the prediction of transmembrane helices in protein structures. His study of the sequence structure relationship shows very limited differences with regards to the structural disagreement. This is very encouraging finding which shows that accurate prediction of TMH could lead to identifying the secondary structure in a protein sequence.

In this paper, we focus on the determination of TMH spanning segments and the amino-terminal orientations. We introduce TMHindex which predicts TMH segments solely from the amino acid sequence information. The prediction is done by using a TMH compositional index which is deduced from the dataset of TMH segments and the amino acid composition. A TMH preference profile is then generated by calculating the average TMH index values along the amino acid sequence using a sliding window of different sizes. Finally, a genetic algorithm was employed to refine the prediction by detecting the optimal set of threshold values that separate the TMH segments from non-TMH segments.

## Materials and Methods

**Figure 1:**
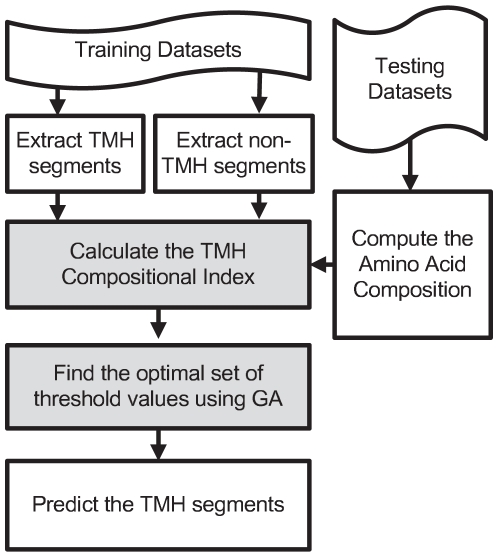
TMHindex overview.

In this section we introduce our method of predicting TMH proteins topology referred to as TMHindex. An overview of TMHindex method is shown in Figure 1. TMHindex consists of the two following major steps which are further detailed in subsequent sections:

1. Calculation of the TMH compositional index: In this step we extract the TMH segments and non-TMH segments from the training dataset, compute the difference in amino acid appearances in TMH segments and non-TMH segments, compute the amino acid composition of the test protein sequence and finally calculate the TMH compositional index.
2. Employing a Genetic Algorithm (GA) to find the optimal set of threshold values: In this step we tailor a GA to find an optimal set of threshold values that will accurately segregate TMH and non-TMH segments.

### TMH compositional index

We start by analyzing the amino acid composition in TMH segments and non-TMH segments. We denote by 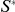 the enumerated set of sequences in the database of membrane protein sequences. From each protein sequence 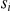 in 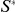, we extract known TMH and non-TMH segments and store them in datasets 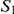 and 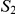, respectively. To represent the preference for amino acid residues in TMH segments, we define an index 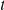. The index 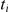 for the amino acid 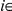 {A, R, N, D, C, Q, E, G, H, I, L, K, M, F, P, S, T,W, Y, V}, is calculated as follows:
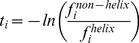
(1)where 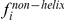 and 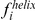 are respectively the frequencies of amino acid 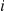 in the datasets 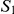 and 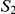. The negative value of 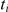 (threshold value of 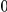) indicates that the amino acid 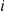 preferably exists in a TMH segment. This is rather analogous to the DomCut method [20] which was developed to predict the inter-domain linker regions in amino acid sequences. However, the information contained in the index values 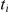 alone is insufficient to accurately predict the TMH segments, thus we incorporated the amino acid composition knowledge to 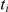 index. The conventional amino acid composition (AAC) values contain 20 components, each of which reflects the normalized occurrence frequency for one of the 20 native amino acids in a sequence. Owing to its simplicity, the AAC model was widely used in many earlier statistical methods for predicting protein attributes. It has also been used in many bioinformatics applications such as inferring the lifestyle of an organism from the characteristic properties of its genome [21] and compensating for the lack of domain information in predicting protein-protein interaction [22].

To this end, we recalculate the compositional index 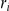 as follows:
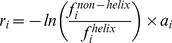
(2)where 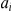 is the AAC of amino acid 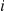. We then represent each residue in all the testing protein sequences by its corresponding compositional index 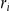. Subsequently, the index values are averaged over a window that slides along the length of each protein sequence. To calculate the averaged compositional index values 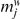 for a protein sequence 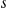, given a single window size 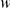, we apply the following formula:
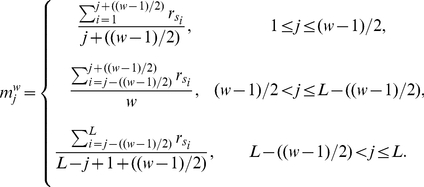
(3)where 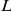 is the length of the protein and 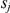 is the amino acid at position 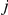 in protein sequence 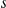.

To illustrate the calculation of the averaged compositional index values 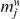, we use the 1LGH:B protein sequence (AERSLSGLTEEEAIAVHDQFKTTFSAFIILAAVAHVLVWVWKPWF). In Table 1, we show the calculation of 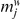 for the first 5 amino acids with a window size 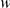 equal to 5.

**Table 1:** Illustration of the calculation of the averaged compositional index values 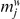.

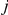	Amino Acid 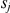	AAC 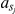	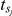	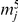
1	A	15.556	−0.30841	(15.556*(−0.30841)+8.889*(1.472438)+2.222*(1.473881))/3 = 4.160103797
2	E	8.889	1.472438	(15.556*(−0.30841)+8.889*(1.472438)+2.222*(1.473881)+6.667*(0.137164))/4 = 3.120077848
3	R	2.222	1.473881	(15.556*(−0.30841)+8.889*(1.472438)+2.222*(1.473881)+6.667*(0.137164)+8.889*(−0.53791))/5 = 1.53976588
4	S	6.667	0.137164	(8.889*(1.472438)+2.222*(1.473881)+6.667*(0.137164)+8.889*(−0.53791)+6.667*(0.137164))/5 = 2.68218555
5	L	8.889	−0.53791	(2.222*(1.473881)+6.667*(0.137164)+8.889*(−0.53791)+6.667*(0.137164)+2.222*(−0.07568))/5 = 0.030853082
:	:	:	:	:

As revealed in the MemBrain method [10], the fusion of various window sizes provides more flexibility in accounting for the length variation of TMHs. This reduces the bias towards a fixed TMH length, introduced by using only one window size (as treated in most of the previous TMH topology predictors). Therefore, averaging is carried across a sequence of odd window sizes ranging from 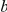 to 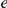 (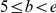), generating a series of features for each protein sequence. This yields the set of values 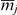 for each sequence:
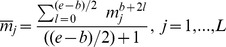
(4)where 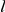 is the summation index that ranges across the 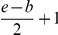 odd window sizes. The values 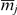 are further used in conjunction with Genetic Algorithm (GA) to refine the prediction by detecting short loops and turns that separate the TMH segments.

### Dynamic threshold using GA

Finding an optimal threshold value which separates TMH segments from non-TMH segments is crucial to the accuracy of the topology prediction. It is a challenging matter that remains unsolved by many existing predictors, most of which use fixed threshold values to separate TMH segments from non-TMH segments (e.g. residues with scores higher than a defined threshold value, are assigned to a helical segment). Indeed, this is a weakness because an optimal threshold for defining two TMH segments separated by long loops is different from a threshold required for identifying TMH segments separated by short loops or tight turns. High-resolution structures show that two consecutive TMH segments are often connected by very short loops or turns and that is why in MemBrain [10] for instance, the authors have utilized a dynamic threshold value in which a base threshold propensity of 0.4 was used to initially define TMH fragments. Then, the threshold was raised according to the shape of the local propensity profile for identifying short loops or helical breaks in fragments. Despite the success shown by utilizing a dynamic threshold, it is noted that raising the threshold could improve the predictions of the TMH segments in part of the sequence and could reduce the prediction accuracy in another part of the sequence.

The prediction problem turns into a search a set of dynamic threshold values that will better reflect the structure of the amino acid sequence and predict accurately the TMH and non-TMH segments. Such a search problem can be viewed as a partition problem [23] which is unsolvable in a polynomial time algorithm. The application of metaheuristic search techniques to this class of problems is a promising solution [23]–[25]. Metaheuristics are high-level frameworks that employ heuristics to find solutions for combinatorial problems at a reasonable computational cost, with strategies ready for adaptation to specific problems. In particular, GA is one of the most commonly used techniques and has proven its effectiveness in combinatorial optimization [23]. Besides, GA is easily customizable for our problem. In the following section we focus on the adaptation of GA to our TMH segment prediction method.

#### Customized Genetic Algorithm

The basic idea of GA is to typically start from a set of initial solutions, and use biologically inspired evolutionary mechanisms to derive new and possibly better solutions [24]. The derivation starts by an initial solution set 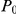 (called the initial population), and generates a sequence of populations 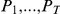, of new solutions applying the genetic operators, crossover and mutation, with probability values 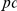 and 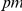, respectively. The 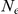 fittest chromosomes of each population are automatically added to the next generation. The algorithm stops if a convergence criterion is satisfied or if a fixed number of generations is reached.

To apply GA to a specific problem, all elements of the generic algorithm must be customized and adapted to the problem. In particular, the solutions must be encoded into chromosomes and the two operators (crossover and mutation) and the fitness function must be defined.

#### Encoding a protein sequence as a chromosome

**Figure 2:**
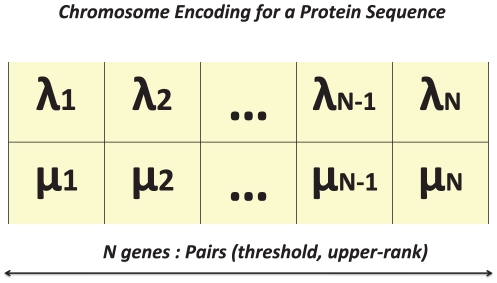
Encoding protein sequence as a chromosome.

To properly apply GA to our problem, we define a chromosome encoding for the protein sequence represented by a vector of 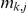 values, calculated using Equation 4. As each chromosome is a set of genes of size 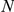, we encode a gene as a pair 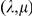, where 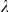 is a threshold value and 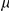 is the upper rank in the protein sequence before which 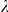 is used as threshold. To illustrate this, let (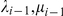), (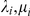) and (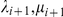) be three consecutive genes in the chromosome representing the sequence of a given protein. The value 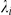 is the threshold applied from the position 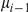 to the position 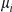 in the protein sequence and 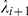 is the threshold applied from the position 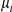 to the position 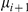 in the sequence. In particular, the threshold 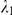 would be applied from the beginning of the sequence to the position 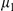 as illustrated in Figure 2.

#### Customized Crossover and Mutation

Based on the chromosome representation and the arithmetic nature of our solution, we define one-cut point crossover. This is a standard way to perform crossover between the chromosomes. It consists of cutting at a position 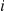 one of the two parent chromosomes into two subsets of genes (vector of pairs 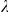 and 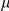). Then the second chromosome is cut at the position 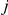 into two other subsets. The cutting point 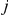 is determined as the rank of the pair (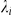, 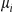) where the position 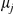 is the smallest position in the second parent chromosome greater than 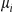. Two new chromosomes are then created by interleaving the subsets.

Mutation is the second reproduction operator that occurs with a small probability 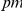. When a chromosome is selected for mutation, a small number of its genes are randomly chosen to be modified. With our chromosome encoding, two ways of modifying a gene (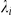, 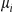) are used. In the first, the threshold 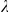 is modified by making a positive or negative variation of its value, while in the second way, the upper bound 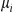 is moved either towards 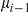 or 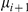.

### Evaluation measures

**Figure 3:**
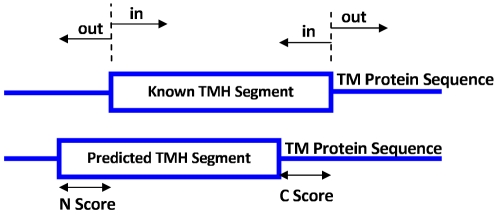
The N and C scores.

To test the TMHindex method and compare its performance to the existing state-of-the-art predictors, we used the following evaluation measures:

1. TMH segment prediction success rate (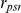),
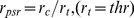
(5)where 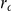, 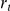 and 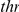 are the number of TMH segments correctly predicted, the total number of TMH segments in the test dataset and the total number of TMH segments, respectively. A prediction is considered correct if there is an overlap of at least nine amino acids between the predicted and the experimentally known TMH segment. This threshold length is quite reasonable compared to the typical TMH which are on average 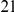 residues long. In the past, various length of residues overlap was used such as 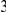 [12], 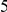 [26] and 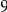 [10].
2. Protein prediction success rate (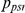),
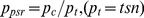
(6)where 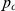, 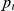 and 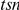 are the number of correctly predicted proteins, the total number of proteins in the test dataset and the total number of testing protein sequences, respectively. A protein is considered correctly predicted if all of its TMH segments are correctly predicted.
3. Amino acid prediction success rate (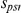),
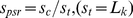
(7)Where 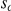 and 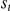 are the number of correctly predicted amino acids and the total number of amino acids in a protein sequence, respectively. This evaluation measure is also used as a fitness function in the proposed GA.
4. The N-score and C-score,  These two scores (illustrated in Figure 3) evaluate the accuracy of predicting the in and out ends of TMHs [27]. N- and C-scores are the number of N- and C-terminal residues that do not match when comparing the predicted TMH segment and the known TMH segment. A lower score in this case means a more accurate prediction. If the prediction of this TMH segment is an exact match, then the N- and C-scores should be equal to 0.
5. Sensitivity (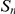) and specificity (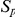),
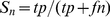
(8)

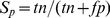
(9)where 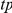 is the number of amino acids within the known TMH segment predicted as ‘TMH’, 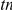 is the number of amino acid out of the known TMH segment predicted as ‘non-TMH’, 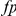 is the number of amino acid out of the known TMH segment predicted as ‘TMH’ and 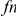 is the number of amino acid within the known TMH segment predicted as ‘non-TMH’.

## Results and Discussion

### Illustration

**Figure 4:**
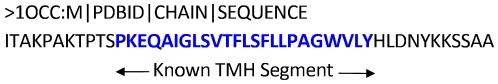
Sample protein 1OCC.

**Figure 5:**
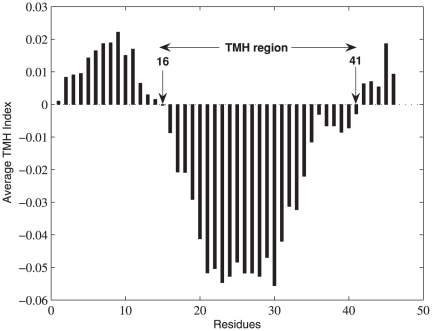
TMH segment detection in protein 1OCC using the index 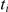.

To illustrate the experimental work, in Figure 4 and Figure 5 we show the way the TMH segment is detected in a sample protein 1OCC using the index 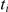 with a threshold value of 0. We used odd window sizes, from 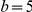 to 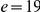, to calculate 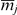 values which represent each amino acid in the sequence. The maximum window size was chosen to be 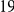 because a 19-residue segment is close to the thickness of the hydrocarbon core of a lipid bilayer [28]. In the sample sequence, the known TMH segment (in bold) starts in residue 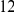 and ends in residue 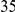. The length of the protein sequence 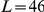 and therefore 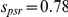, C-score = 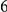 and N-score = 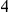.

**Figure 6:**
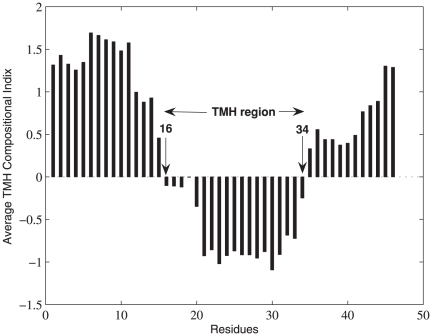
TMH segment detection in protein 1OCC using the compositional index 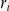.

To improve the prediction accuracy we incorporated the compositional index 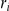 and the results are shown in Figure 6, where we can easily spot the improved accuracy, i.e., 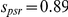, C-score = 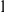 and N-score = 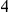.

**Figure 7:**
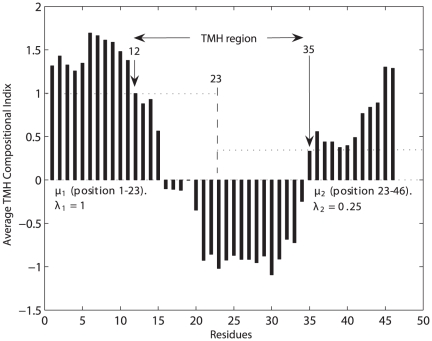
TMH segment detection in protein 1OCC using GA.

As a second enhancement of our approach, GA was applied to find the optimal threshold set separating TMH segments from the non-TMH segments, as illustrated in Figure 7. Prior to the application of GA, several runs were performed to tune the different parameters. As a result of parameter tuning, the number of generations 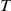 was set to 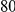 and the population size to 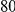. During the reproduction process, crossover and mutation occur with probabilities 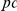 equal to 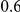 and 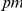 equal to 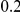, respectively. The elitism strategy was used by which the 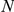 fittest chromosomes of one generation are cloned and copied to the next generation. After applying GA to the sequence of the protein 1OCC, the latter is divided into 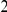 equal parts. Each part consists of 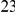 residues and the two upper boundary positions, 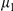 and 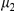, are respectively found by GA to be 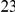 and 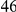. The threshold values 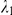 and 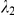 are computed to be 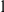 and 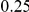, respectively. The obtained structure of the protein 1OCC, as computed by GA, achieved high accuracy, i.e., 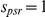, C-score = 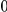 and N-score = 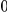.

### Comparison with existing methods

The aim of the TMH segment prediction method is to obtain high accuracy when applied to unknown proteins. For predicting the TMH segment within a protein, we first computed the index 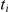. We collected the TMH and non-TMH segments from a training dataset. The training dataset contains 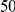 protein sequences which consist of 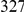 known TMH segments. The testing dataset contains 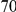 protein sequences which consist of 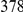 known TMH segments. The training and testing datasets have experimentally determined TMH topology and were used by most of the available TMH predictors such as MemBrain [10], Phobius [14], THUMBU [13] and TMHMM [12]. The datasets are available at http://faculty.uaeu.ac.ae/nzaki/TMHindex.htm.

The performance of TMHindex was measured by 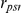, 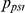, N-score, C-score and the number of TMH segments which were correctly predicted. The comparison of the performance of TMHindex against those of THUMBU, SOSUI, DAS-TMfilter, TOP-PRED, TMHMM, Phobious and MemBrain, are reported in Table 2. The results show that TMHindex is successful in making fewer mis-classifications of TM helices. It outperforms the compared methods according to all of the measures used for performance evaluations. To analyze the performance of TMHindex based on approximately one helical turn, we calculated 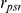 based on an overlap of five amino acids between the predicted and the experimentally known TMH segment. The accuracy of 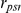 in this case was found to be 100%.

**Table 2:** Performance comparison of various TMH predictors.

Predictor	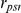 (%)	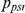 (%)	N-Score	C-Score	Correct TMHs
THUMBU	85.5	47.1	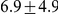	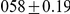	316
SOSUI	89.1	57.1	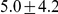	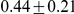	334
DAS-TMfilter	90.7	64.3	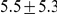	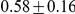	341
TOP-PRED	92.6	60	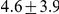	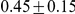	352
TMHMM	91	65.7	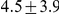	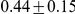	343
Phobious	91.8	71.4	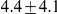	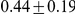	345
MemBrain	97.9	87.1	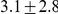	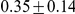	371
TMHindex	99.46	91.1	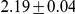	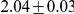	376

TMHindex was able to predict 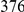 of the total 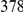 TMH segments in the testing dataset. The unpredicted TMH were from proteins 2IUB:A and 2B5F:A. Furthermore, the amino acid prediction success rate in terms of 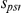, 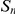 and 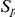 were 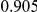, 0.901 and 0.865, respectively.

**Figure 8:**
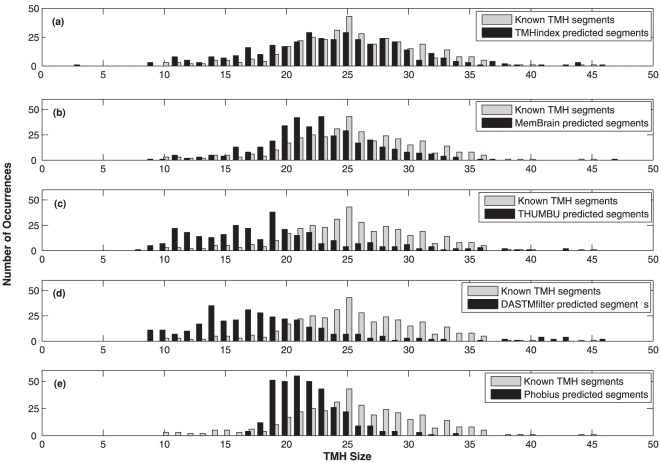
Length distribution of the 378 known TMHs in the testing dataset compared to predicted TMHs using (a) TMHindex, (b) MemBrain, (C) THUMBU, (d) DAS-TMfilter and (e)Phobius methods.

The distributions of helix lengths in the testing datasets were also examined (Figure 8). This is an essential feature because there is a wide distribution of TMH length amongst the 70 helical polytopic membrane proteins in the testing dataset. Our method in this case demonstrated significant ability in correctly identifying the ends of TMHs. The investigation shows that the prediction methods typically search for TM helices with length ranging between 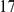 and 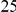 residues. In fact, out of the 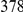 TM helices in the dataset, only 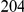 (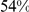) of the helices fall within this range, 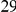 (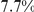) have length less than 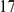 and 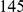 (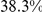) of the helices have over 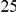 residues. Several membrane proteins contain TM helices that do not span the bilayer. For example, the pore (P) helix of the potassium channel KcsA (1K4C) and the nitropropionic acid (NPA) contain loops of the aquaporins. These ‘half-TMs’ are shorter in length than conventional TM helices and are expected to be more difficult to predict [27]. The distributions of TM helices given in Figure 8 reveal a small but significant population of half-TMs to be present in the testing dataset. Similarly, there are many TMH segments which are longer than 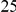 residues in length that often ended unpredicted or partially predicted by most of the available methods. Figure 8 clearly shows that Phobius is unable to detect TMH segments shorter than 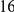 and longer than 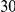 residues. DAS-TMfilter and THUMBU are unable to detect many TMH segments longer than 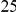 residues. MemBrain is unable to detect many TMH segments longer than 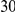 residues. The only remark that needs more inversigation of the TMHindex method is related to the prediction of some TMH segments of length 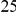. Their predictions show more errors than any other segments.

For further validation, TMHindex was also tested on 73-protein 3D helix database created by Zhou et al. [13]. The dataset was used to assess the predictions of THUMBU method [13]. Pylouster et al. [19] have also used 56 proteins with correct resolutions out of the 73 proteins to study the influence of assignment on the prediction of TMH in protein structure. The percentage of proteins with correct TMH segments (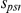) predicted using TMHindex was 91.8%. The prediction accuracy in this case is superior to the accuracy acheived by other methods such as THUBMU (87.7%), TOP-PRED II (68.5%), TMHMM 2.0 (68.5%) and MEMSAT 1.8-3D (84.9%) reported by Zhou et al. [13]. Furthermore, 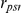, 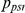, N-score, C-score, 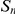 and 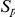 were 0.987, 0.922, 2.007, 1.517, 0.905 and 0.901, respectively.

The accuracy achieved using TMHindex in comparison to the known methods for predicting the topology of TM proteins is a strong indication of its capability. The performance of the proposed method is due to two main reasons. The first one is the employment of the TMH compositional index, which was deduced from a dataset of prior known TMH segments and the incorporation of the amino acid composition knowledge. The second one is tailoring GA, which offered a flexible way to model an intelligent predictor of TM proteins topology based on more dynamic thresholds.

The current version of TMHindex needs appriximately 20 minutes for predicting and converging towards accurate structures of the available 70 protein sequences using a computer equipped with Intel Core 2 Duo CPU T7250 @ 2.00 GHz and 2.99 GB of RAM.

In the future, we will extend the TMHindex method to predict signal peptides. Predicting TMH and signal peptides is challenging because of the high similarity between the hydrophobic regions of a TMH and that of a signal peptide [14]. Although, the GA customization has significantly improved the prediction, further tuning and other strategy choices within the metaheuristic framework could achieve more capable and flexible prediction.
